# RDAD: A Machine Learning System to Support Phenotype-Based Rare Disease Diagnosis

**DOI:** 10.3389/fgene.2018.00587

**Published:** 2018-12-04

**Authors:** Jinmeng Jia, Ruiyuan Wang, Zhongxin An, Yongli Guo, Xi Ni, Tieliu Shi

**Affiliations:** ^1^The Center for Bioinformatics and Computational Biology, Shanghai Key Laboratory of Regulatory Biology, The Institute of Biomedical Sciences and School of Life Sciences, East China Normal University, Shanghai, China; ^2^Beijing Key Laboratory for Pediatric Diseases of Otolaryngology, Head and Neck Surgery, The Ministry of Education Key Laboratory of Major Diseases in Children, Beijing Pediatric Research Institute, Beijing Children's Hospital, Capital Medical University, National Center for Children's Health, Beijing, China; ^3^National Center for International Research of Biological Targeting Diagnosis and Therapy/Guangxi Key Laboratory of Biological Targeting Diagnosis and Therapy Research/Collaborative Innovation Center for Targeting Tumor Diagnosis and Therapy, Guangxi Medical University, Nanning, Guangxi, China

**Keywords:** rare disease, phenotype, machine learning, diagnostic model, web-based tools

## Abstract

DNA sequencing has allowed for the discovery of the genetic cause for a considerable number of diseases, paving the way for new disease diagnostics. However, due to the lack of clinical samples and records, the molecular cause for rare diseases is always hard to identify, significantly limiting the number of rare Mendelian diseases diagnosed through sequencing technologies. Clinical phenotype information therefore becomes a major resource to diagnose rare diseases. In this article, we adopted both a phenotypic similarity method and a machine learning method to build four diagnostic models to support rare disease diagnosis. All the diagnostic models were validated using the real medical records from RAMEDIS. Each model provides a list of the top 10 candidate diseases as the prediction outcome and the results showed that all models had a high diagnostic precision (≥98%) with the highest recall reaching up to 95% while the models with machine learning methods showed the best performance. To promote effective diagnosis for rare disease in clinical application, we developed the phenotype-based Rare Disease Auxiliary Diagnosis system (RDAD) to assist clinicians in diagnosing rare diseases with the above four diagnostic models. The system is freely accessible through http://www.unimd.org/RDAD/.

## Introduction

Rare diseases are rare conditions that occur only in a precious few people. Currently, there is no unified, widely accepted definition for rare diseases (Jia and Shi, 2017). To facilitate increased communication, knowledge sharing and coordinated orphan drug development across national borders, the World Health Organization (WHO) defines rare diseases as a prevalence >6.5–10 in 10,000 (Franco, 2013), which we adopted as the definition of rare diseases in this article. About 80% of rare diseases are the consequence of genetic defects, but >5% of rare diseases can be effectively interfered with or treated. Nowadays, screening and diagnostic rates of rare diseases are constantly improved with the progress of molecular biology and cytogenetics (Ekins, 2017). For example, whole-exome sequencing has allowed for the discovery of the genetic cause for a considerable number of diseases, opening up new ways for disease diagnostics, especially for OMIM (Online Mendelian Inheritance in Man) disorders. However, due to the lack of clinical samples and records, the molecular causeremains difficult to identify (Qi et al., 2017; Wu et al., 2017). Therefore, only a limited number of rare Mendelian diseases can be diagnosed through DNA sequencing, making clinical phenomic information a major resource to diagnose rare diseases (Jia and Shi, 2017). Disease phenotypes (also known as clinical phenotypes) refer to the observable characteristics of an organism (or cell), including individual form, function and other aspects of performance, such as height, color, blood type and enzyme activity. Usually, phenotypes associated with rare diseases are described by a set of clinical medical terms. To provide better interoperability in the field of rare diseases, several tools have been specifically designed to assist in standardizing, and sharing of clinical medical terms, through various medical resources (Dragusin et al., 2013; Girdea et al., 2013; Yang et al., 2015; Maiella et al., 2018). For example, Phenomizer aims to help diagnose genetic diseases from the input list of symptoms and PhenoTips provides a framework to share and analyze patient data between professionals. At present, the main approach to support disease diagnosis is based on disease similarities calculated from diseases' clinical phenotypes, using a semantic hierarchy of the Human Phenotype Ontology (HPO; Alves et al., 2016). Under this circumstance, the similarity score between two diseases will be highly dependent on the completeness and specificity of their annotated phenotypes. To overcome the limitations, we adopted both the traditional phenotypic similarity method and a new machine learning method to build four diagnostic models to support the diagnosis of rare diseases. We then validated the performance of all these models using the real electronic medical records (EMR) from RAMEDIS. To promote effective diagnosis of rare diseases in a clinical application, we developed the phenotype-based Rare Disease Auxiliary Diagnosis system (RDAD) to assist clinicians in diagnosing rare diseases using the above four diagnostic models.

## Materials and Methods

**Figure 1:**
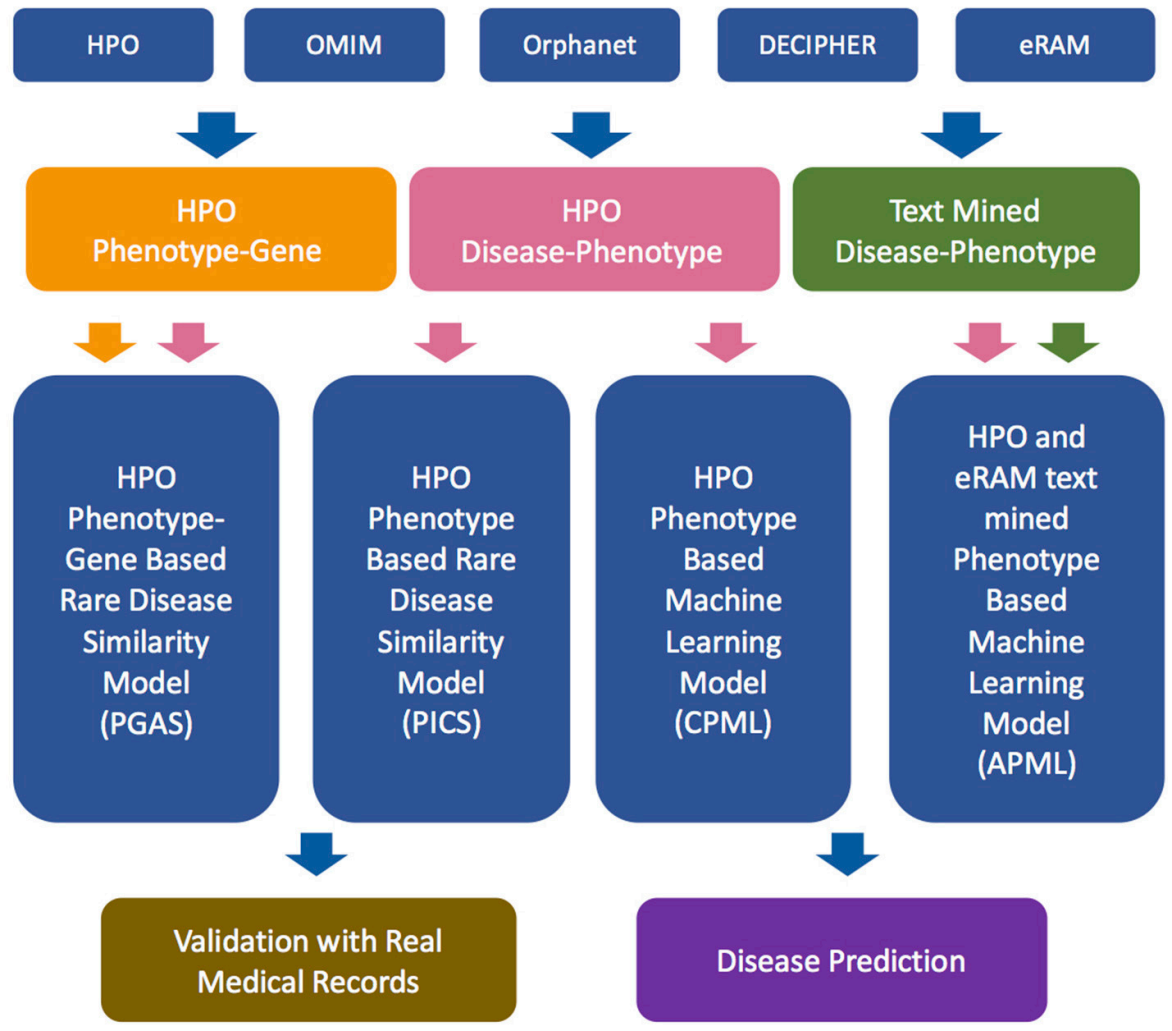
The workflow of RDAD. HPO, Human Phenotype Ontology. OMIM, Online Mendelian Inheritance in Man. PGAS, Phenotype-Gene Association based rare disease similarity model; PICS, Phenotypic TF-IDF-Hierarchy information content based rare disease similarity model; CPML, Curated feature Phenotype spatial vector based rare disease Machine Learning prediction model; APML, Curated and text mined feature phenotype spatial vector based rare disease Machine Learning prediction model.

The workflow of the RDAD is depicted in Figure 1. The data sets for the four diagnostic models contained in the RDAD were integrated from eRAM (Jia et al., 2018a), Human Phenotype Ontology (Robinson et al., 2008), Orphanet (Pavan et al., 2017), OMIM (Amberger et al., 2014), and DECIPHER (Firth et al., 2009). To integrate multi-level biomedical resources and multiple classifiers, we built four diagnostic models. The phenotype based rare disease similarity (PICS) model used curated rare disease-phenotype associations as the input data and four disease similarity methods as the classifiers, while the phenotype-gene based rare disease similarity (PGAS) model used curated rare disease-phenotype associations and curated phenotype-gene associations as the input data and two disease similarity methods as the classifiers. In contrast, the phenotype based machine learning (CPML) model used curated rare disease-phenotype associations as the input data and six machine learning algorithms as the classifiers; similarly, the curated and text-mined phenotype based machine learning (APML) model used curated rare disease-phenotype associations and text mined (Xu et al., 2013) rare disease-phenotype associations as the input data and six machine learning algorithms as the classifiers. The four different diagnostic models contained in the RDAD system, with their input data sets and classifiers are listed in Table 1.

**Table 1:** The Four Diagnostic Models Contained in the RDAD System.

**Data sources**	**Model**			
	**PICS**	**PGAS**	**CPML**	**APML**
HPO Phenotypes	√	√	√	√
eRAM Curated Genes		√		
eRAM Text Mined Phenotypes				√
Disease Similarity Classifiers	√	√		
Machine Learning Classifiers			√	√

### Extraction of Phenotypes and Corresponding Genes

Rare disease names were extracted from eRAM, the rare disease-phenotype associations were extracted from HPO and eRAM, rare disease related genes were mainly collected from eRAM. eRAM is a standardized system that covers a variety of rare diseases, integrates current existing data on clinical manifestations (symptoms and phenotypes) and molecular mechanisms of rare diseases systematically, revealing many novel associations between rare diseases (Jia et al., 2018a). The HPO is a system providig a standardized vocabulary of phenotypic abnormalities that are encountered in human disease (Robinson et al., 2008). We first obtained rare disease names from eRAM, then extracted curated rare disease-phenotype associations from the annotation files (phenotype_annotation_hpoteam.tab, #1249) provided by HPO, which contains annotations made explicitly and manually by the HPO-team (mostly referring to OMIM entries). In addition, we retrieved the rare disease-phenotype pairs from eRAM in which the related records were extracted from abstracts and full-text articles in MEDLINE, through a pattern-based relationship extraction approach (Xu et al., 2013). In total, 8,488,796 abstracts and 774,514 full-text articles were text-mined, respectively, from PubMed and PubMed Central, which lead to the identification of 23,231 rare disease-phenotype pairs.

### Electronic Health Records

RAMEDIS (Rare Metabolic Diseases Database) provides an accurate curated resource of human variations with corresponding phenotypes for rare metabolic diseases (Topel et al., 2010). So far, 93 different genetic metabolic diseases among 818 patients have been released. PhenoTips is an open source framework for analyzing phenotype information for patients with genetic diseases (Girdea et al., 2013). We downloaded all 1,099 medical records from RAMEDIS, and then obtained 818 related records according to the mapping between the diagnostic disease names of medical records (rare disease names were standardized by eRAM). According to the historical description and symptom fields in the medical records, the corresponding phenotypic data from the medical records were extracted with the open source software PhenoTips. Finally, 309 phenotypes were obtained, involving 27 rare diseases, which were subsequently used as the real medical records-based test set for the four different diagnosis models contained in RDAD. The test set extracted from RAMEDIS is listed in Table 2.

**Table 2:** The Test Data Set for the Four Diagnostic Models.

**Diagnosis**	**Case count**
PHENYLKETONURIA (MIM 261600)	157
CONGENITAL DISORDER OF GLYCOSYLATION, TYPE Ia (MIM 212065)	27
MAPLE SYRUP URINE DISEASE (MIM 248600)	21
PROPIONIC ACIDEMIA (MIM 606054)	16
CANAVAN DISEASE (MIM 271900)	15
SUCCINIC SEMIALDEHYDE DEHYDROGENASE DEFICIENCY (MIM 271980)	10
ALKAPTONURIA (MIM 203500)	10
ARGININOSUCCINIC ACIDURIA (MIM 207900)	9
ISOVALERIC ACIDEMIA (MIM 243500)	7
CYSTINURIA (MIM 220100)	5
CITRULLINEMIA, TYPE II, NEONATAL-ONSET (MIM 605814)	5
WILSON DISEASE (MIM 277900)	4
HOLOCARBOXYLASE SYNTHETASE DEFICIENCY (MIM 253270)	4
FANCONI-BICKEL SYNDROME (MIM 227810)	2
ALPHA-METHYLACETOACETIC ACIDURIA (MIM 203750)	2
TYROSINE TRANSAMINASE DEFICIENCY (MIM 276600)	2
HYPERINSULINEMIC HYPOGLYCEMIA, FAMILIAL, 2 (MIM 601820)	2
HAWKINSINURIA (MIM 140350)	2
OSTEOGENESIS IMPERFECTA, TYPE I (MIM 166200)	1
GLYCOGEN STORAGE DISEASE VI (MIM 232700)	1
N-ACETYLGLUTAMATE SYNTHASE DEFICIENCY (MIM 237310)	1
REFSUM DISEASE (MIM 266500)	1
KRABBE DISEASE (MIM 245200)	1
LEIGH SYNDROME (MIM 256000)	1
GLYCOGEN STORAGE DISEASE Ib (MIM 232220)	1
PYRUVATE CARBOXYLASE DEFICIENCY (MIM 266150)	1
PEARSON MARROW-PANCREAS SYNDROME (MIM 557000)	1

### The PICS Diagnostic Model

The input data of the PICS diagnostic model were the curated rare disease-phenotype associations, and we selected the curated phenotypes as the features. Cosine similarity is defined as the evaluation of the similarity between two vectors by calculating the value of the angle cosine. The similarity between the two vectors of the same vector cosine is 1, and the similarity of the two vectors at 90 degrees is 0. If the two vectors are the opposite, the similarity is −1. Cosine similarity is used in the positive space and the value is to be neatly bound in [0,1] (Jia et al., 2018b). Given two feature phenotype spatial vectors, *D* = (*p*_1_, *p*_2_, …, *p*_*n*_), *Q* = (*q*_1_, *q*_2_, …, *q*_*n*_), the cosine similarity is represented using a dot product and magnitude as follows:

$$\begin{array}{l} Cosine\_similarity\;=\frac{D*Q}{\left\|D\|*\|Q\right\|}\\ \;=\frac{\sum_{i=1}^{n}\left(D_{i}*Q_{i}\right)}{\sqrt{\sum_{i=1}^{n}{\left(D_{i}\right)}^{2}}*\sqrt{\sum_{i=1}^{n}{\left(Q_{i}\right)}^{2}}} \end{array}$$

The Tanimoto coefficient is extended by the Jaccard coefficient. Given two feature phenotype spatial vectors, *D* = (*p*_1_, *p*_2_, …, *p*_*n*_), *Q* = (*q*_1_, *q*_2_, …, *q*_*n*_), the Tanimoto coefficient is calculated as follows:

$$Tanimoto(D,Q)=\frac{D*Q}{\|D{\|}^{2}+\|Q{\|}^{2}-D*Q}$$

To provide an antidiastole and to rank the candidate rare diseases in descending order of probability, the score is calculated as follows (Pinol et al., 2017):

$$\Psi_{i}=1-\frac{n}{Max\left[P_{u},P_{i}\right]}$$

Where *P*_*u*_ indicates the phenotypes provided by the user, *P*_*i*_ indicates the phenotypes of rare diseases in the training set, the function of *Max*[*P*_*u*_, *P*_*i*_] refers to the largest number between *P*_*u*_ and *P*_*i*_. *n* signifies the number of different phenotypes between the phenotypes associated with any rare disease in the RDAD database and the phenotypes submitted by the user.

The similarity between two phenotypes can be calculated by the “information content” of their MICA (Most Informative Common Ancestor; Kohler et al., 2009). For each of the phenotypes submitted by the user, the best matched phenotype among the phenotypes related to the rare disease is found, and the average value over all the query phenotypes is then calculated. The similarity is calculated as follows:

$$Similarity\;(Q\to D)=avg\left[\sum_{p_{1}\in D}\underset{p_{2}\in Q}{\max}\;IC\left(MICA\left(p_{1},p_{2}\right)\right)\right]$$

The symmetric version of the above equation is:

$$\begin{array}{l} {Similarity}_{symmetric}\;(D,Q)=\frac{1}{2}\;Similarity\;(Q\to D)\\ \;+\frac{1}{2}\;Similarity\;(D\to Q) \end{array}$$

Based on the TF-IDF-Hierarchy information content (van Driel et al., 2006) matrix of rare disease associated phenotype spatial vector obtained from Data Set I, we used the above methods to construct the PICS model.

### The PGAS Diagnostic Model

The input data of the PGAS diagnostic model were the curated rare disease-phenotype associations and the curated phenotype-gene associations, and we selected the curated genes and curated phenotypes as the features.

Given two feature gene spatial vectors, *G* = (*g*_1_, *g*_2_, …, *g*_*n*_), *Q* = (*q*_1_, *q*_2_, …, *q*_*n*_), the cosine similarity, is represented using a dot product as follows:

$$\begin{array}{l} Cosine\_similarity\;=\frac{G*Q}{\left\|G\|*\|Q\right\|}\\ \;=\frac{\sum_{i=1}^{n}\left(G_{i}*Q_{i}\right)}{\sqrt{\sum_{i=1}^{n}{\left(G_{i}\right)}^{2}}*\sqrt{\sum_{i=1}^{n}{\left(Q_{i}\right)}^{2}}} \end{array}$$

Given two feature gene spatial vectors, *G* = (*g*_1_, *g*_2_, …, *g*_*n*_), *Q* = (*q*_1_, *q*_2_, …, *q*_*n*_), the Tanimoto coefficient, is represented as follows:

$$Tanimoto(G,Q)=\frac{G*Q}{\|G{\|}^{2}+\|Q{\|}^{2}-G*Q}$$

Given two phenotype sets, *P*_1_ = (*p*_1_, *p*_2_, …, *p*_*m*_), *P*_2_ = (*p*_1_, *p*_2_, …, *p*_*n*_), the similarities between two phenotype sets are defined as follows:

$$\begin{array}{l} {Similarity}_{symmetric}\left(P_{1},P_{2}\right)=\frac{1}{2}\;Similarity\;\left(P_{1}\to P_{2}\right)\\ \;+\frac{1}{2}\;Similarity\;\left(P_{2}\to P_{1}\right) \end{array}$$

Based on the rare disease associated phenotype-gene spatial vector obtained from Data Set II, we used the above methods to construct the PGAS model.

### The CPML Diagnostic Model and the APML Diagnostic Model

The input data of the CPML diagnostic model were the curated rare disease-phenotype associations, and we selected the curated phenotypes as the features. Similarly, the input data of the APML diagnostic model were the curated rare disease-phenotype associations and the text mined rare disease-phenotype associations, and we selected the curated phenotypes and text mined phenotypes as the features.

Based on the TF-IDF-Hierarchy information content matrix of rare disease associated phenotype spatial vector obtained from Data Set III and Data Set IV, the CPML model, and the APML model take classifier performance into consideration. We first adopted Logistic Regression, KNN, Random Forest, Extra Trees, Naive Bayes, and Deep Neural Network machine learning classification algorithms as classifiers, respectively, and then used the Bayesian averaging algorithm in both models to leverage the prediction results of the six classifiers, ranking candidate rare diseases by their scores.

### The Data Sets for the Four Diagnostic Models Contained in the RDAD System

The training sets for the four diagnostic models contained in the RDAD system are listed in Table 3. All rare diseases in the four training data sets were regarded as model labels. The phenotypes in Data Set I/III/VI were used to calculate the phenotypic TF-IDF-Hierarchy information content, based on the phenotype semantic hierarchy of HPO. The genes in Data Set II were used to calculate phenotype similarity and the phenotypes in Data Set II were used to calculate the rare disease similarity based on the phenotype similarity in the PGAS model. The records in Data III/IV were used as the input data for the machine learning classifiers in the CPML model and the APML model.

**Table 3:** The Training Data Sets for the Four Diagnostic Models.

**Data set**	**Model**	**Term**	**Term count**
Data Set I	PICS	Rare Diseases	4,498
		Curated Phenotypes	5,990
		D-P Associations	57,346
Data Set II	PGAS	Rare Diseases	4,498
		Curated Phenotypes	5,990
		D-P Associations	57,346
		Curated Genes	3,682
		P-G Associations	419,597
Data Set III	CPML	Rare Diseases	4,498
		Curated Phenotypes	5,990
		D-P Associations	57,346
		Synthetic Patients	44,980
Data Set IV	APML	Rare Diseases	4,498
		All Phenotypes	6,453
		D-P Associations	72,404
		Synthetic Patients	44,980

*D-P Associations, Disease-Phenotype association pairs; P-G Associations, Phenotype-Gene association pairs*.

### The Four Diagnostic Models in the RDAD System With Their Corresponding Classifiers

To facilitate rare disease diagnosis, we applied the phenotypic TF-IDF-Hierarchy information content on the phenotype semantic hierarchy of Human Phenotype Ontology (HPO), and then built the phenotypic TF-IDF-Hierarchy information content based on the rare disease similarity model (PICS), the phenotype-gene association based rare disease similarity model (PGAS), and the curated feature phenotype spatial vector based rare disease machine learning prediction model (CPML), as well as the curated and text mined feature phenotype spatial vector based rare disease machine learning prediction model (APML). The four diagnostic models contained in RDAD with their corresponding classifiers are listed in Table 4.

**Table 4:** The Four Diagnostic Models with Their Corresponding Classifiers.

**Model**	**Data set**	**Classifier**	**Score**
PICS	Data Set I	Cosine Similarity	Bayesian Averaging Algorithm
		Tanimoto Coefficient	
		Ψ_i_ Score	
		MICA	
PGAS	Data Set II	Cosine Similarity	Bayesian Averaging Algorithm
		Tanimoto Coefficient	
CPML	Data Set III	Logistic Regression	Bayesian Averaging Algorithm
		K-Nearest Neighbor	
		Random Forest	
		Extra Trees	
		Naive Bayes	
		Deep Neural Network	
APML	Data Set IV	Logistic Regression	Bayesian Averaging Algorithm
		K-Nearest Neighbor	
		Random Forest	
		Extra Trees	
		Naive Bayes	
		Deep Neural Network	

*MICA, Most Informative Common Ancestor*.

### Precision and Recall

Precision measures the fraction of correct predictions made by the four diagnostic models contained in the RDAD system. Recall (or specificity) measures the fraction calculated by dividing the number of correct choices by the total number of choices available to each model. True positives (TP) are the number of correctly predicted rare diseases, false positives (FP) are the number of incorrectly predicted rare diseases and false negatives (FN) are the number of rare diseases that are not predicted. The F1-score is an aggregate measure for the accuracy of a classifier that calculates a weighted average of Precision and Recall defined as follows (Alves et al., 2016):

$$\begin{array}{l} Precision\;=\frac{TP}{TP+FP}\\ \;Recall\;=\frac{TP}{TP+FN}\\ F1_{-}Score\;=\frac{\;Precision\text{*}Recall}{2*(\;Precision\;+\;Recall\text{) }} \end{array}$$

## Results

### Precision and Recall

**Figure 2:**
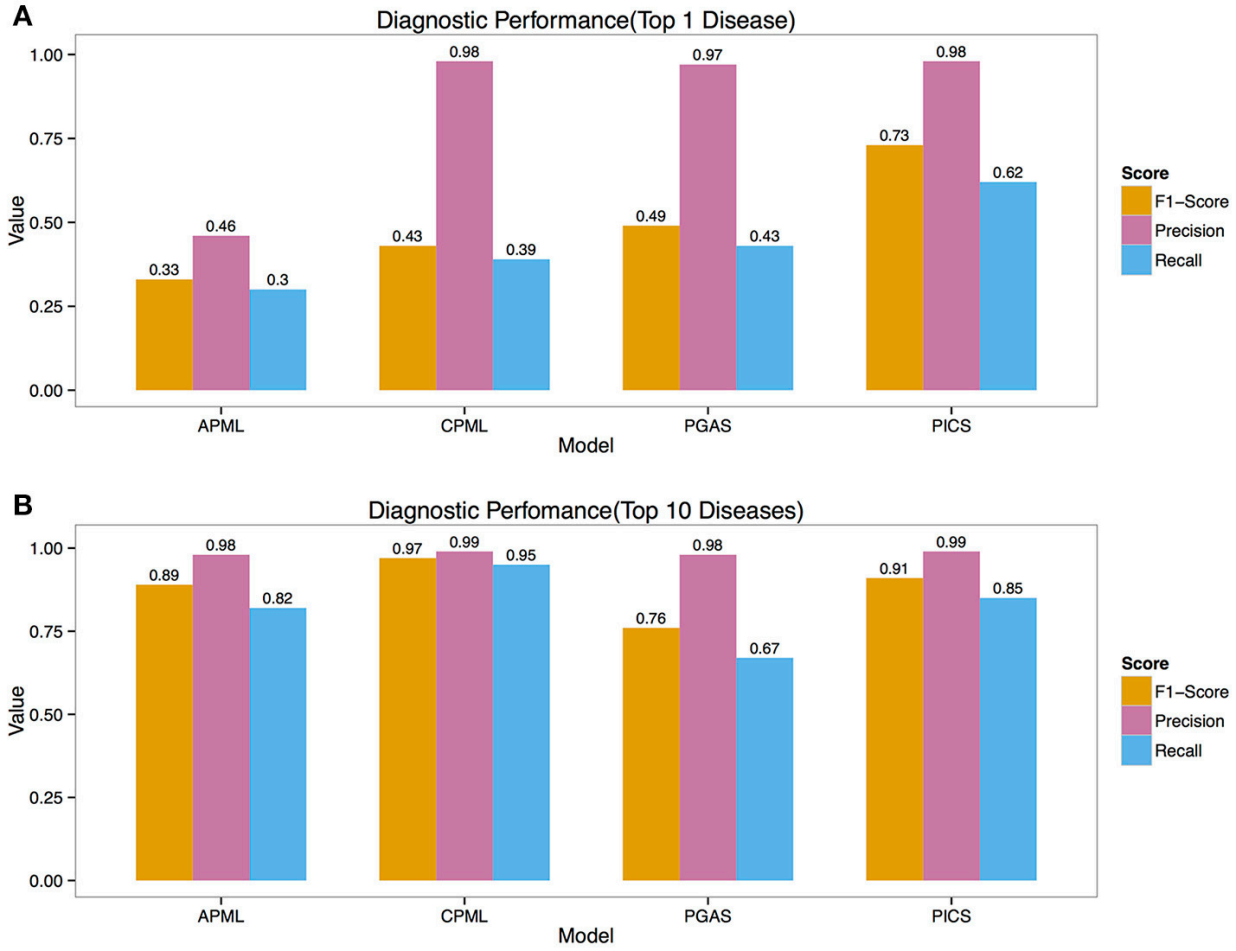
The Precision, Recall, F1-Score of Different Models. (**A)** The top 1 diagnostic performance. (**B)** The top 10 diagnostic performance. APML, the curated and text mined feature phenotype spatial vector based rare disease machine learning prediction model. CPML, the curated feature phenotype spatial vector based rare disease machine learning prediction model. PGAS, the phenotype-gene association based rare disease similarity model. PICS, the phenotypic TF-IDF-Hierarchy information content based rare disease similarity model.

**Figure 3:**
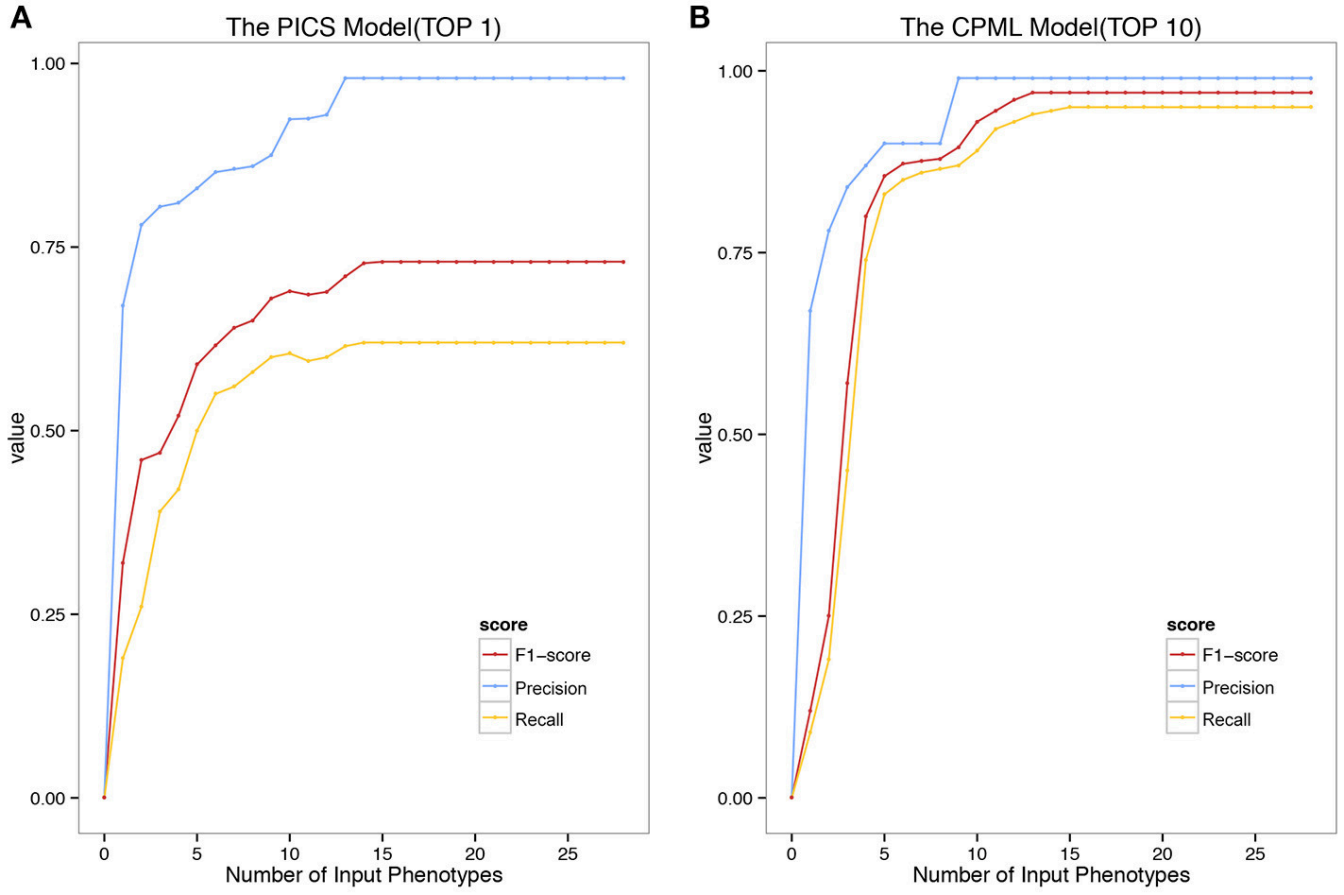
The Precision Recall and F1-Score of the model with different number of Phenotypes Submitted. (**A)** The top 1 diagnostic performance of PICS model. (**B)** The top 10 diagnostic performance of CPML model.

We validated the above four models with the real medical records from RAMEDIS. The results showed that the PICS model achieved the best performance among the four models, with only one rare disease as the outcome of the prediction (Figure 2A), but in real application, the diagnosis result is barely satisfactory. To better help clinicians pinpoint the right disease, we then provided a credible list of the top 10 candidate diseases as the prediction outcome, which will help clinicians narrow down candidate diseases through the diagnostic process. Under such circumstances, the CPML model had the best performance (Figure 2B). In addition, in order to achieve the best result for rare disease diagnosis, RDAD suggests that the number of inputted phenotype terms of each selected diagnostic model is around 15 (Figure 3). The average number of symptoms recorded in the EMR in RAMEDIS database was 17, indicating that the suggested number of the RDAD model (around 15) is feasible.

### Confusion Matrix

**Figure 4:**
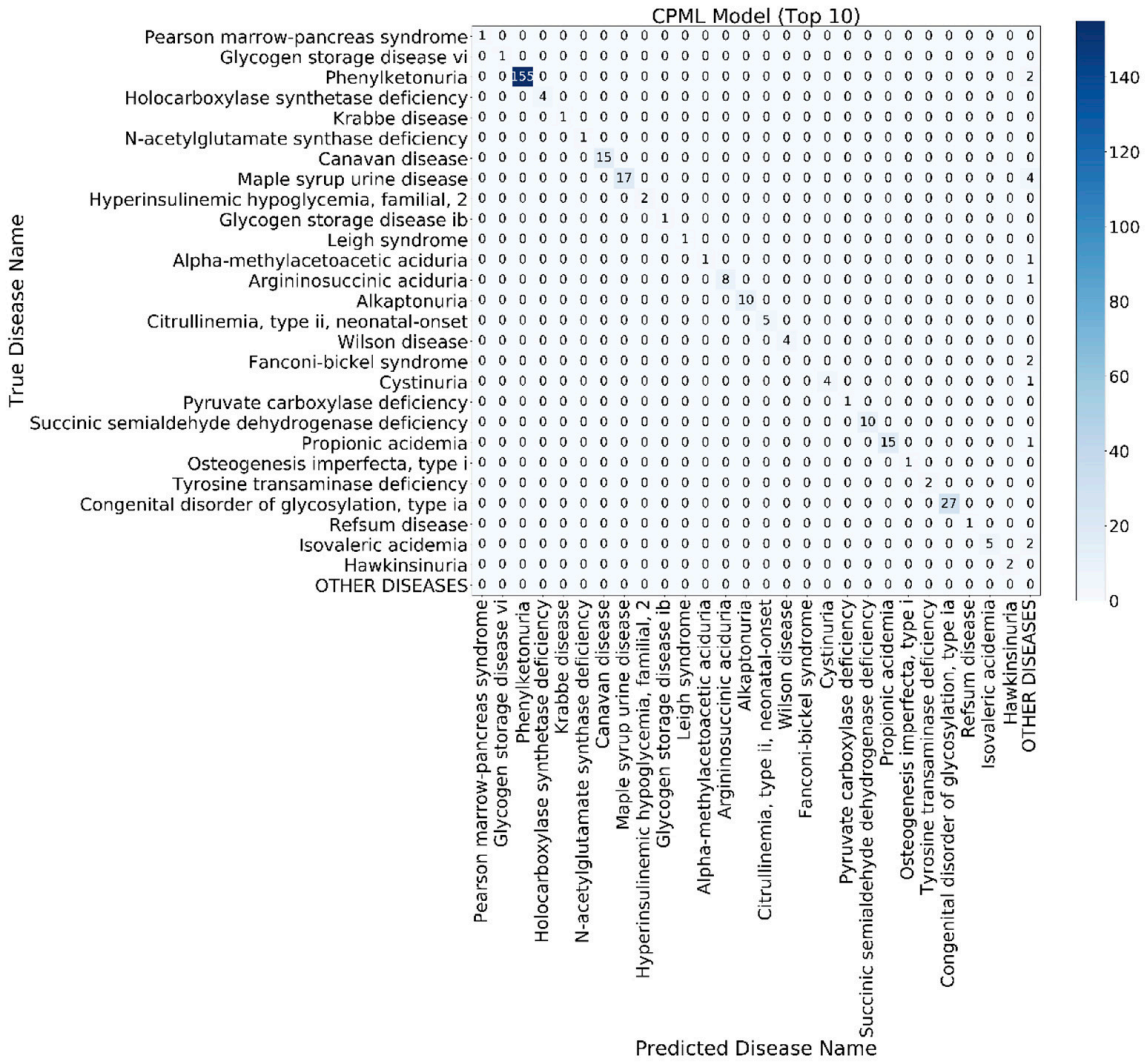
The top 10 candidate rare diseases confusion matrix of the CPML model. The ylab refers to the disease names of the records, while xlab refers to the candidate disease names provided by the diagnostic model.

The confusion matrix is a special two-dimensional contingency table with the same class set on two dimensions. We built a confusion matrix of the top 10 rare disease candidates for each model using the EMR from RAMEDIS. The confusion matrixes of different models showed that machine learning diagnostic models (CPML and APML) performed better than traditional disease similarity models (PICS and PGAS). Compared to other models, the CPML model showed the best performance (Figure 4, Figure S1).

### Candidate Rare Diseases Rank

**Figure 5:**
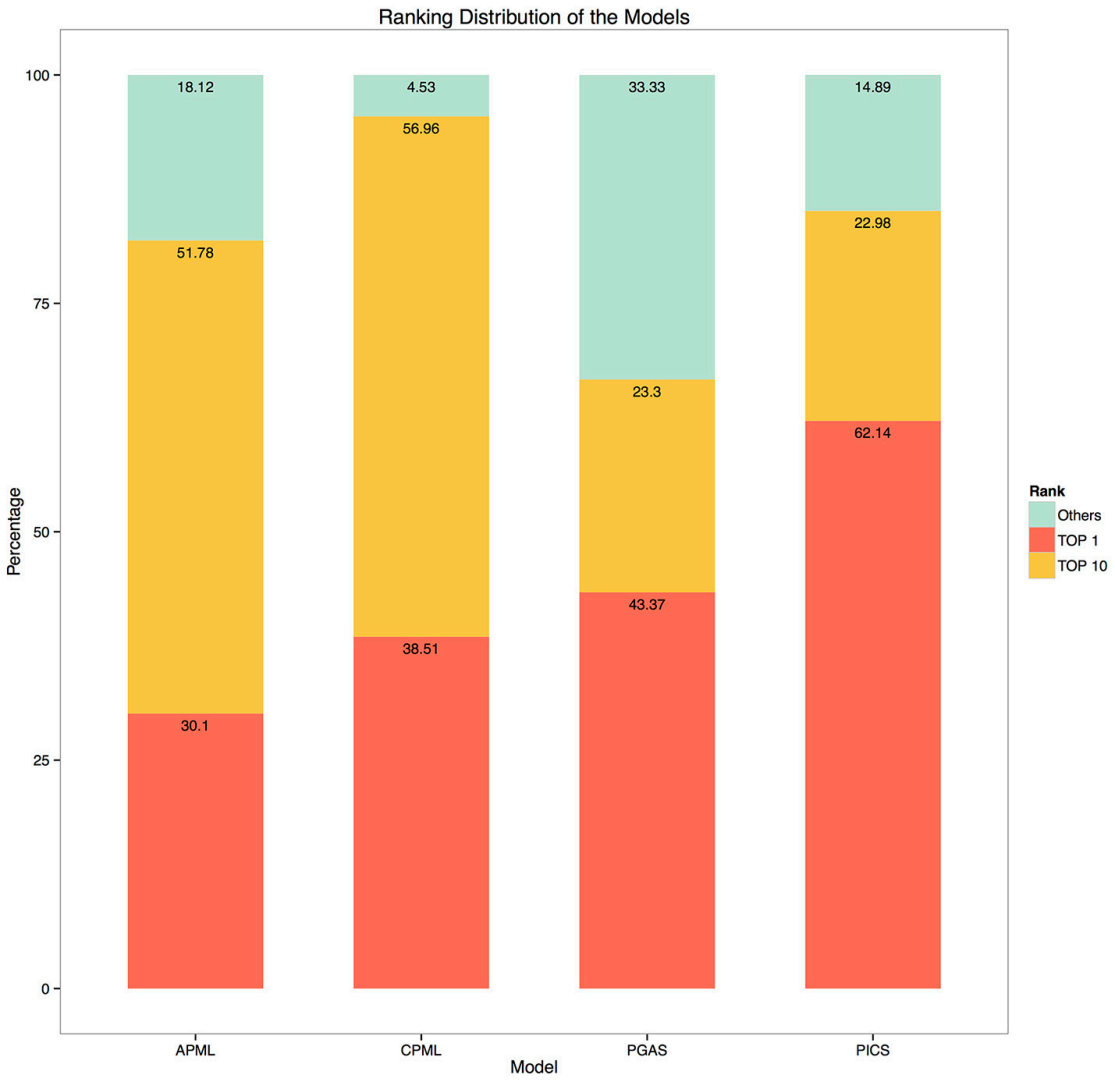
The ranking distribution of the models. The ylab refers to the percentage of disease rankings, while xlab refers to the diagnostic models.

Given the input phenotypes, we examined the candidate rare diseases detected, ranked as top 1, top 10 and others with the four diagnostic models in RDAD. We found that 62.1% of the designated rare diseases were ranked as top 1 with the PICS model, the good performance of this model is most likely due to the accuracy of the associated phenotype of rare diseases and the direct calculation between the spatial vectors while the other three models undergo a series of transformations during data processing, resulting in information loss and error amplification. In contrast, 95.5% of the correct rare diseases were ranked as top 10 with the CPML model. Thus, our results clearly demonstrate that the four diagnostic models contained in the RDAD system are suitable for finding rare diseases that are known to be associated with phenotypes. In general, the model built by the machine learning method, showed better performance. The four diagnostic models successfully ranked the most likely candidate rare diseases in the top 10 (Figure 5).

Compared with the above results (Figures 2, 3), the result showed that the performance of the classifiers varied in different cases, but where similar to ensemble learning (ensemble learning is a machine learning paradigm where multiple learners are trained to solve the same problem). In contrast to ordinary machine learning approaches that try to learn one hypothesis from training data, ensemble methods try to construct a set of hypotheses and combine them. After using the Bayesian averaging algorithm in the four models to integrate the prediction results of their classifiers, ranking candidate rare diseases with a score, classification results of the four diagnostic models were stable. At the same time, the accuracy and recall rate ranked at the top, varied significantly in the four models built by different data or classifiers. A possible reason for this could be that every patient more or less presents some noise phenotypes and many rare diseases have similar phenotypes, which can interfere with the prediction of the correct rare disease. However, misclassification is significantly reduced when the top 10 is selected as the cutoff value for the predictive outcome, which represents an improvement of the reliability of the model results and is also the designated value we recommend during real application.

## Discussion

Rare diseases always have a wide range of complex and diverse phenotypes. However, clinicians always lack knowledge on rare diseases or clinical experiences. Many rare diseases can therefore not be accurately identified on time, and patients are most likely to not receive an accurate diagnosis and subsequent effective treatment. Moreover, due to the heterogeneity of rare diseases, the lack of available clinical diagnostic tests also hinders the timely diagnosis of corresponding diseases. Computer assisted decision support tools have been introduced since the 1960s (Warner, 1989), after which many algorithms were introduced, such as Bayes classifiers (Trace et al., 1990), neural networks (Barnett et al., 1987), rule-based systems (Miller, 1986), and Bayesian networks (Schurink et al., 2005). In this article, we described both the disease similarity method and the machine learning method based diagnostic models for rare disease. We clearly noticed that classifier performance varied in different cases, but similar to ensemble learning, after adopting the Bayesian averaging algorithm in the four models, integrating the prediction results of their classifiers and ranking the candidate rare diseases with score. At the same time, the accuracy and recall rates for all four models built by different data or classifiers, changed significantly when ranked as the top condition, while robustness was ensured when ranked in the top 10 conditions. The reason for this could be that each patient will present some “noise phenotypes,” which might interfere with the classification of the model.

Like all the other computer aided diagnosis tools, any rare disease not included in the corresponding model training set cannot be predicted by each diagnostic model contained in the RDAD. In addition, the limited real data sets (EHR/EMR) and diverse patients in this study also restrict the performance of the models. At present, although users are strongly recommended to choose the CPML model in the RDAD system to assist rare disease diagnosis, the RDAD still provides all 4 diagnostic models as alternative to rare disease diagnosis. On the one hand, although the current result show that machine learning models perform better than disease similarity models, PICS performs the best in ranking the top condition (F-1 score 0.73, Precision 0.98 and Recall 0.62). On the other hand, the CPML model performs better than the APML model, but the diagnosis can only be reliable when candidate diseases have corresponding phenotypic annotation in the HPO. For diseases that only have text mined phenotypes, APML will be a better choice; therefore, the four different models can complement each other under different circumstance. It is anticipated that with the accumulation of clinical phenotypes of rare diseases, the performance of our models will improve gradually.

## Author Contributions

TS supervised the study. JJ and ZA designed and developed the diagnostic models. RW and ZA built up the website. YG and XN verified the validation results. JJ wrote the manuscript. All authors have read and approved the final manuscript.

### Conflict of Interest Statement

The authors declare that the research was conducted in the absence of any commercial or financial relationships that could be construed as a potential conflict of interest.
