# Supplementary material for: RDAD: A Machine Learning System to Support Phenotype-Based Rare Disease Diagnosis
Source: Front Genet. 2018 Dec 4;9:587. doi: 10.3389/fgene.2018.00587 (PMC6288202; doi:10.3389/fgene.2018.00587)
Supplement: Supplementary file 1 [file Data_Sheet_1.DOCX]

## Supplementary Material 1

## ROC & AUC

We used ROC curves and AUC values to evaluate each classifier. Phenylketonuria (MIM:261600), a rare disease with the largest number of associated medical records, was tested as an example in nine machine learning classifiers. The ROC curve of phenylketonuria classifiers can be seen in Fig S1. Our results demonstrate that classifier performance varies in different cases.

**
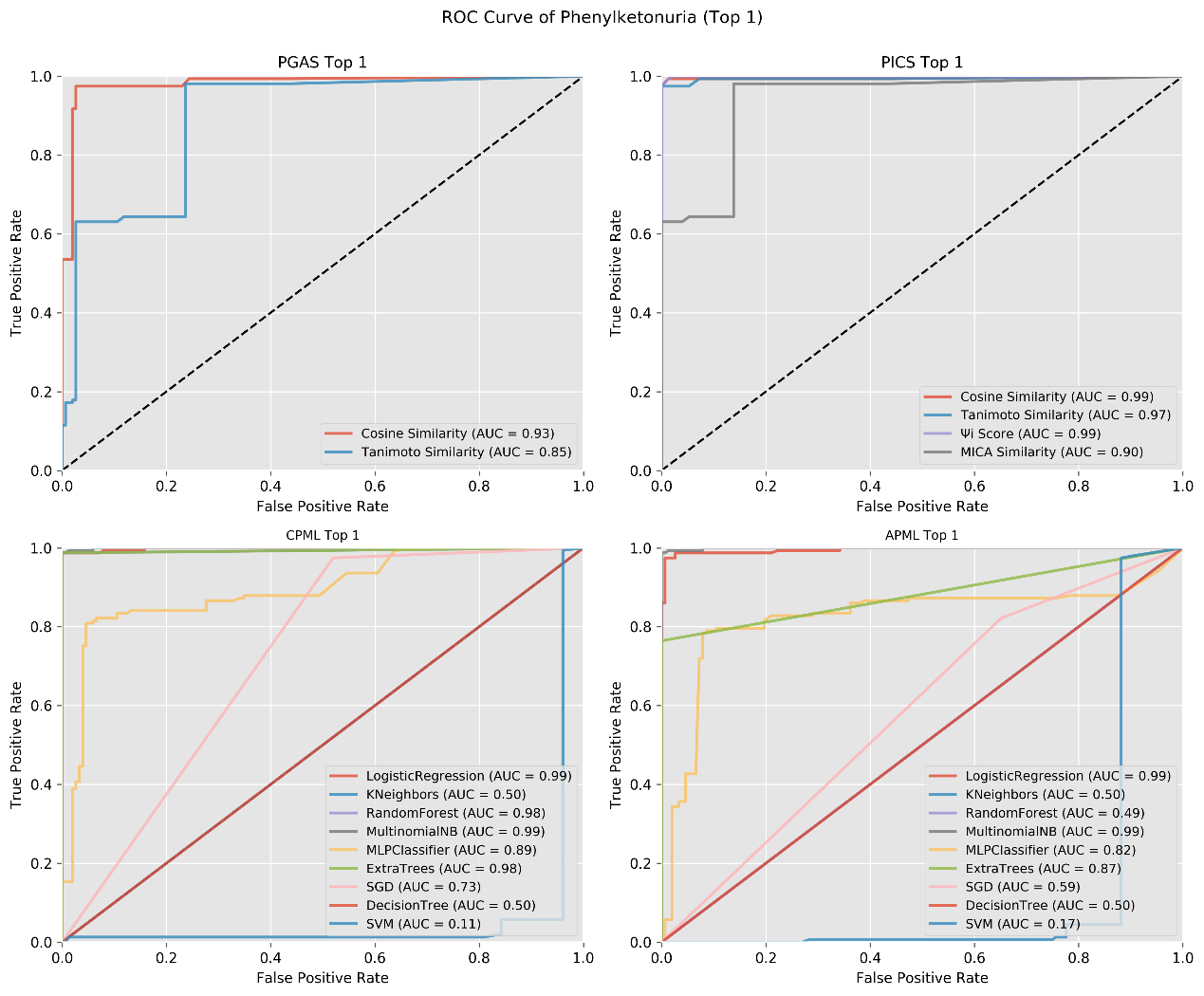
**

**Fig. S1.** The ROC Curve of Phenylketonuria Classifiers. **(a)** The phenotypic TF-IDF-Hierarchy information content-based rare disease similarity model. **(b)** The phenotype-gene association-based rare disease similarity model. **(c)** The curated feature phenotype spatial vector-based rare disease machine learning prediction model. **(d)** The curated and text-mined feature phenotype spatial vector-based rare disease machine learning prediction model. **APML**: Curated and text-mined feature phenotype spatial vector-based rare disease machine learning prediction model. **CPML**: Curated feature phenotype spatial vector-based rare disease machine learning prediction model. **PGAS:** Phenotype-gene association-based rare disease similarity model. **PICS**: Phenotypic TF-IDF-Hierarchy information content-based rare disease similarity model.
