# Supplementary figures and images for: RDAD: A Machine Learning System to Support Phenotype-Based Rare Disease Diagnosis
Source: Front Genet. 2018 Dec 4;9:587. doi: 10.3389/fgene.2018.00587 (PMC6288202; doi:10.3389/fgene.2018.00587)

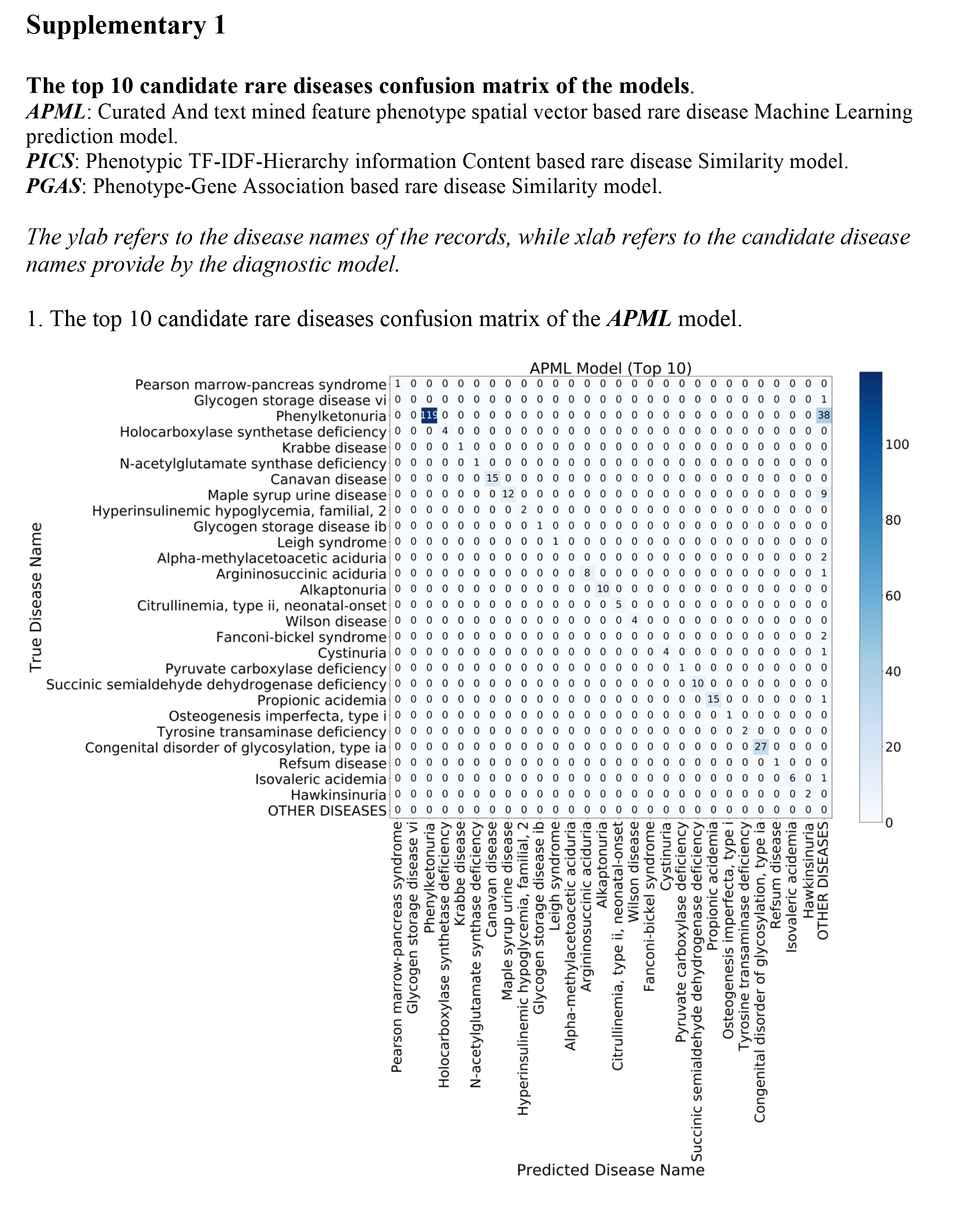

Supplement: Supplementary file 2 [file Image_1.PNG]

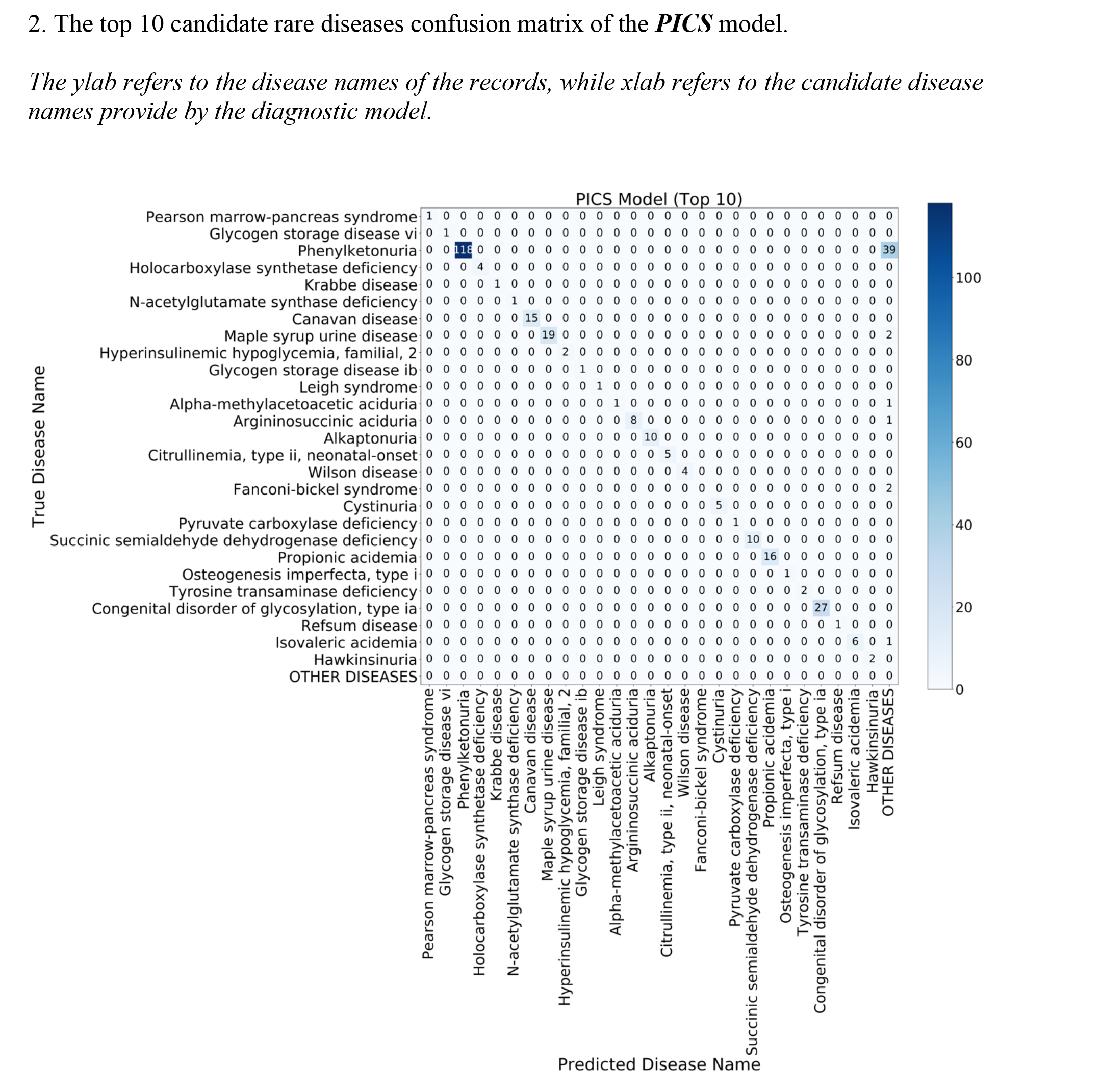

Supplement: Supplementary file 3 [file Image_2.PNG]

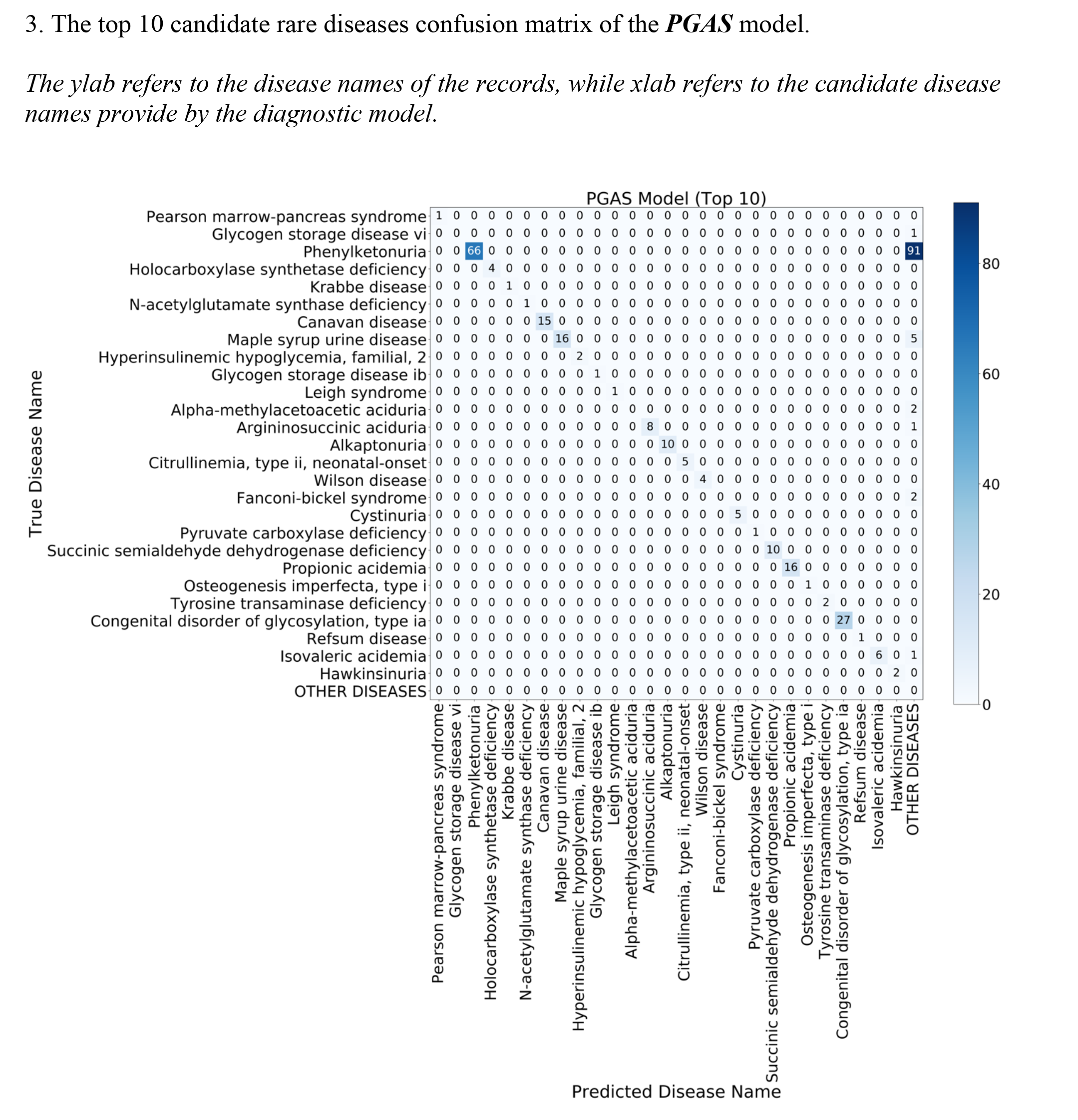

Supplement: Supplementary file 4 [file Image_3.PNG]
